# ClimActor, harmonized transnational data on climate network participation by city and regional governments

**DOI:** 10.1038/s41597-020-00682-0

**Published:** 2020-11-06

**Authors:** Angel Hsu, Zhi Yi Yeo, Ross Rauber, James Sun, Yunsoo Kim, Sowmya Raghavan, Nicholas Chin, Vasu Namdeo, Amy Weinfurter

**Affiliations:** 1 Yale-NUS College, 16 College Ave W, Singapore, 138609 Singapore; 2 University of Chicago, Department of Computer Science, 5730 S. Ellis Ave, Chicago, IL 60637 USA; 3 Yale College, Department of Statistics and Data Science, 24 Hillhouse Ave, New Haven, CT 06511 USA

**Keywords:** Climate-change mitigation, Politics

## Abstract

Cities and regions have become increasingly engaged in global climate change governance. They are pledging their own climate mitigation targets and participating in membership networks that typically are transnational in nature and engage thousands of subnational governments. Researching these growing trends in participation has been difficult due to the disparate and inconsistent nature of this self-reported data. To facilitate future analyses of these actors, we introduce ClimActor, the largest harmonized global dataset of more than 10,000 city and regional governments participating in networks like the Global Covenant of Mayors for Climate and Energy, C40 Cities for Climate Leadership, ICLEI Local Leaders for Sustainability, among others. We include key contextual information on each actor’s population, geographic location, and administrative jurisdiction to facilitate disambiguation of potential overlaps in actions or emissions. We also provide a series of cleaning functions based on phonetic and fuzzy string matching algorithms within an open-source R package to make it easy for anyone to immediately use the ClimActor dataset with other relevant data.

## Abstract (summary)

**Table:** 

Measurement(s)	climate action network participation
Technology Type(s)	digital curation • computational modeling technique
Sample Characteristic - Environment	city
Sample Characteristic - Location	Earth (planet)

Machine-accessible metadata file describing the reported data: 10.6084/m9.figshare.12994214

## Background & Summary

Cities and regions- defined as subnational administrative units generally broader in population and area than cities and the first administrative level below national governments^1^ - are increasingly engaged in global climate change governance. They hold the majority of the world’s population and are at high risk for experiencing the impacts of climate change^2^. These subnational actors are pledging their own largely voluntary climate actions, which include setting emissions reduction targets, increasing renewable energy consumption, and financing for energy efficiency and other technology upgrades^3^. While some of these actions support implementation of national climate policies^4^, many subnational actors participate in transnational climate initiatives that engage actors working across borders towards shared goals^5,6^, and often in collaboration with national governments^1^. The EU Covenant of Mayors for Climate and Energy, for example, includes more than 9,000 primarily small cities in Europe (population less than 50,000) to commit to emission reduction targets more ambitious than the European Union’s own pledges^7^. Participating in these networks creates opportunities for actors to enter into dialogues with other cities and to pool their global influence as critical sustainability actors^8,9^. They can also provide benefits spanning agenda setting, information sharing, and capacity building^10^. Cities may join several initiatives to participate in multiple dialogues with slightly different participants, focus areas, or reputational benefits, particularly if the cost of entry or membership is low.

While the number of cities and regions participating in these transnational climate networks has risen dramatically over the last few decades^11,12^, analyzing their collective impact has been challenging, largely due to lack of available data on the impact of subnational actors’ climate actions and incomplete and heterogenous data, when they are available^13,14^. A recent survey of subnational climate actors that participate in reporting platforms found that just 58 percent had sufficient information to quantify their potential mitigation impact^15^. Further complicating the aggregation and estimation of these actors’ contributions to global climate mitigation is the difficulty in determining overlaps between actors: city governments embedded within states often commit their own climate actions without coordinating with higher levels of jurisdictions, which could result in double counting of emissions reductions or other activities if not appropriately accounted for^14,16,17^. Due to these challenges, most studies evaluating subnational climate actions are focused on a small number of cities with available data^18,19^ or localized to a specific region^20^.

**Fig. 1:**
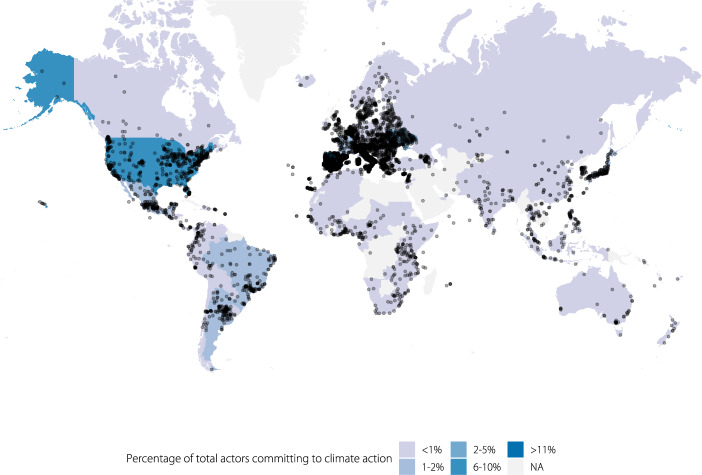
Global distribution of subnational actors committing to climate action platforms included in the ClimActor dataset and R package.

**Fig. 2:**
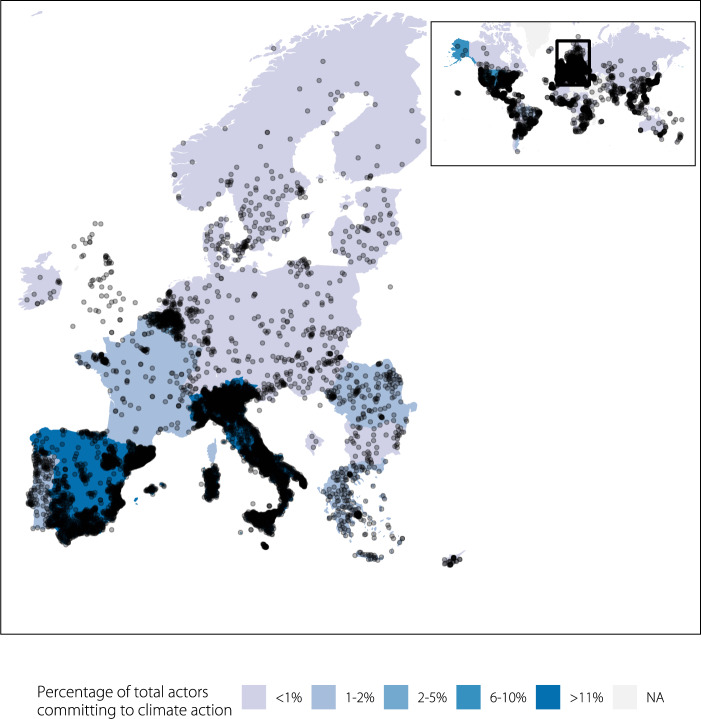
Most subnational actors committing to climate action in the ClimActor dataset are concentrated in Europe, with Italy representing the highest number of actors (n = 4,201).

**Fig. 3:**
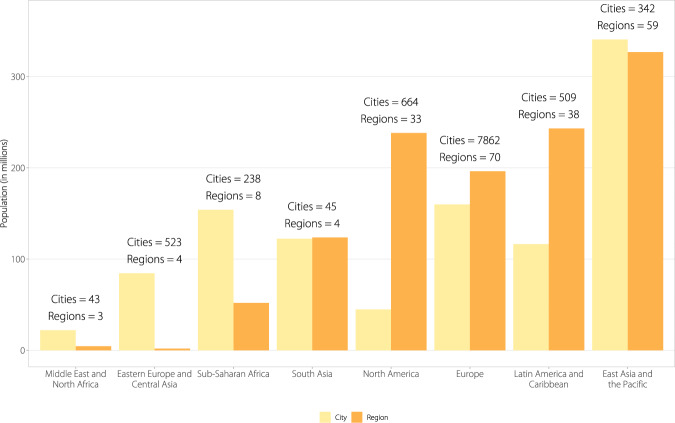
Population of subnational actors (cities and regions) recording climate action commitments as captured in the ClimActor dataset, grouped by region.

To obtain a full global picture of which subnational governments are taking climate actions as reported through primarily transnational climate initiatives, such as the Global Covenant of Mayors for Climate and Energy, C40 Cities for Climate Leadership, and ICLEI Local Leaders for Sustainability, this paper shares what we believe to be the most comprehensive and harmonized database of subnational climate action network participation and contextual information to date. It is intended to underpin research efforts to build on and extend analyses on the scope and impacts of subnational climate action. To create it, we collated data from multiple sources (see Online-only Table 1) and developed an R package (“ClimActor”) that provides a series of functions to aid in cleaning, matching and mapping these disparate datasets and enables users to match this information with other datasets (i.e., on emissions, commitments, etc.) in a standardized way. The ClimActor subnational climate action dataset includes more than 10,000 subnational actors’ location, population, area and participation in climate action networks (Figs. 1–3).

The ClimActor dataset helps navigate several key challenges and considerations around the analysis of subnational climate action. Data platforms often represent subnational actors in disparate ways: New York City, for example, can appear as New York, NY, New York, or the city of New York on multiple platforms but refer to the same city of more than 8 million people. Collecting information on which actors have signed up to various pledging platforms and then merging these data with critical information on population or emissions is then challenging because actor names must be cleaned and standardized to ensure actors’ data are correctly matched. Actor names can be misspelled or subnational governments’ could have similar names (i.e., London could refer to London, United Kingdom or London, Ontario or even London, Kentucky). The process of disambiguating these names and matching datasets can be incredibly laborious and time-consuming^21^. To address these obstacles, the ClimActor dataset compiles data on subnational climate network participation and relevant contextual data, enabling researchers to explore how climate action participation and trends vary within and across different geographic and economic contexts (Fig. 3)^15,22^.

The dataset also addresses the challenges of disentangling overlaps and hierarchies in subnational climate action data. Identifying overlaps between different levels of jurisdiction and avoiding instances of “double-counting” is a vital part of many climate action analyses^14^. Identifying the links between local, regional, and national governments also aids efforts to explore the nuances of subnational climate action within specific national governance contexts^6^. The ClimActor database lists the networks an actor participates in as well as its higher-levels of political administration, making it possible to understand overlaps in climate commitments. This information can be used to avoid double-counting commitments reported across several different platforms and to facilitate explorations of subnational engagement with transnational climate initiatives. It can also help determine what commitments are not subsumed within a nested jurisdiction but instead in addition to (i.e., “additional”) to what a higher level of government has pledged, if any (i.e., if New York City and New York State both make climate pledges, understanding their nested relationship would allow for an analyst to make an evaluation of whether actions at the lower level are truly in addition to what a higher level of administration has pledged)^1^.

## Methods

### Participation data

Membership data for subnational actors participating in climate action networks (Online-only Table 1) such as the carbon*n* Climate Center and EU Covenant of Mayors for Climate and Energy initiatives and UNFCCC’s Global Climate Action Portal were collected from the respective websites using Python v3.6.5^23^ and the BeautifulSoup v4.6^24^ and json^25^ packages. Generally, the workflow to collect actor data from these websites is as follows: a complete list of actors within the database is first extracted from the site and if other relevant information is available for an actor (e.g., location, population, etc.), that information is collected and matched to an actor’s name. Data are then cleaned using the pandas package in python^26^ before being stored as csv files. Online-only Table 1 lists the complete climate action networks that were collated for the ClimActor dataset.

### Contextual data

If a climate action platform or database does not include contextual information on a particular actor, such as population, location, area, etc., this data is collected from Wikipedia’s English language pages. Although a crowdsourced website and information repository, Wikipedia has been shown to be an accurate source of political data and information^27^. Wikipedia is accessed through the MediaWiki API using Python, and the BeautifulSoup, json, and requests^28^ packages. Each actor in the database is minimally associated with a name, entity type (either City, Region, Company, or Country), and ISO country code, each of which is used to resolve which Wikipedia page corresponds to an actor in question. This procedure begins by passing an actor’s name to the MediaWiki API’s search function, which returns a list of potential matches ordered by relevance. The top 20 pages are each downloaded and prepared for further filtering by mapping each page to a likely entity type and country.

To ensure the correct Wikipedia page is selected for a subnational actor, the Wikipedia categories to which a page belongs are fed to a function that counts the occurrences of certain words that likely correspond to a specific entity type (e.g., words like municipality, town, capital, ward, and village are more commonly found in pages that should be in the City category). If the word “disambiguation” occurs in a page’s categories, the page is taken to be a Disambiguation page^29^. For instance, an actor in question that could have a name that refers to something else (e.g., Golf, Illinois could be mistaken for the sport of golf if no other contextual information is included). Otherwise, the entity type with the highest count of matching words is then taken to be the page’s entity type. If no words match, the page is assigned no entity type.

To infer a page’s country, the page’s “info-box”–the light grey table on the right of many Wikipedia pages–is searched for links to Wikipedia pages corresponding to the countries on the site’s list of countries assigned an ISO code (ISO, 2020). The first of these links found in the infobox is taken to be the one corresponding to the country the page should be assigned to. If no links are found within the infobox, the page’s body text is then searched according to the same procedure.

The list of 20 search results is then filtered such that only pages matching the actor in entity type and ISO code remain. The first of these pages–deemed by the Wikipedia search function to be most relevant–is then selected as the best candidate and is mined for contextual information according to the procedure described in the following paragraph. If no pages remain after filtering the top 20 for entity type and ISO code, the results are checked for any disambiguation pages. If one is found, then each of the links in the disambiguation page are processed and filtered as if they were the results of the search above, the first matching page being selected if multiple matches are found. If still no matches are found, then the process is repeated starting from the call of the search function including an actor’s ISO code in the search query. If still no matches are found, the actor is not resolved to a page.

If a page is found during this process, contextual information is extracted from the page’s info-box, a table containing rows usually several sections indicated by a single column spanning the entire table, each of which contains several related rows with two columns, the left one describing the data found in the right column. For example, under the section “population,” a row may be found containing two columns with values “Metro” and some number respectively. This information is used to find relevant population and figures, matched with corresponding years where possible. In addition to this data, latitude and longitude are collected in decimal degrees where possible. To account for potential overlaps between city and region actors, the latter which often encompass the former, we also collect information on city actors’ higher jurisdictions. For example, Manhattan (borough) is located in New York City (city) within New York (state). This information can be used to avoid double counting of climate actions, although there are some manual disambiguations that are still needed. Wallonia Region in Belgium, for instance, is a higher administrative unit than other regional actors, such as Antwerp Province and Brussels Capital Region, and encompasses five other provinces within Belgium (Liège, Hainaut, Luxembourg, Walloon Brabant, and Namur). Avoiding overlaps between Wallonia Region and other city or municipality actors within Belgium would need to account for overlaps within these provinces located in Wallonia as well. In some cases, regional councils commit to climate action and encompass several regions and cities, such as the Assembly of Regions of Cote d’Ivoire (ARDCI).

Because we only scraped English-language Wikipedia pages, we acknowledge that some contextual information is missed through Wikipedia pages in other languages (see Technical Validation:Data Limitations). Other data sources for country-level contextual data (i.e., population, land area) were obtained from the sources listed in Online-only Table 2.

## Data Records

The ClimActor package includes three main datasets: subnational climate action participation and accompanying contextual information for each actor (stored as “contextuals”); a key dictionary (“key_dict”) of subnational actor standardized and unstandardized names for purposes of string matching (see ‘Usage Notes’ below); and a country dictionary (“country_dict”) for cleaning country names associated with each actor that is required for the cleaning functions in the ClimActor package. The datasets are available on figshare^30^, and are also stored as Rdata objects and automatically loaded when the ClimActor package is loaded within a user’s R environment. They are also available as CSV files and can be downloaded from the ClimActor package’s GitHub page (www.github.com/datadrivenenvirolab/ClimActor). An overview of the included information within the datasets is provided in Online-only Table 2.

## Technical Validation

We performed validation checks primarily on the self-reported population and geographic location data from subnational actors via climate action reporting platforms. Platforms such as CDP and the Global Covenant of Mayors for Climate and Energy include population data, although sometimes the year associated with the data are not included and require validation. Because actors may simultaneously report to multiple climate action platforms at once and report conflicting data, we compared and validated the data to ensure the database reflects the most recent and accurate data available. Below we describe the validation checks applied to self-reported population and geographic location data.

### Population data

In instances where subnational actors report to multiple climate action platforms and different population data are collected from these various sources, we compared the self-reported population numbers with each other. If the values varied within five percent of each other, we selected the first entry of that actor within the database. If the values varied more than five percent of each other, we prioritized the following data sources’ population data: 1) CDP (formerly known as the Carbon Disclosure Project), as they require cities and regions to report on an annual basis; 2) Global Covenant of Mayors for Climate and Energy or EU Covenant of Mayors for Climate and Energy; 3) Carbon*n* Climate Registry. For actors in which no population data was reported to these three sources, population data was obtained from Wikipedia. For actors where population data was available but where the year corresponding to the population data was missing, the year corresponding to the population data was estimated by comparing the population data with the actor’s Wikipedia page. For example, if an actor’s population data matched the number on its Wikipedia page, we imputed the corresponding population year with the year listed on an actor’s Wikipedia page.

### Geographic location data

In instances where a subnational actors’ geographic location data (longitude, latitude) were provided, we validated by plotting all actors’ coordinates on a map (Fig. 1). A global polygon of countries’ boundaries were used to identify potentially erroneous coordinate information. Outliers were then visually inspected and the geographic coordinate information was removed and replaced with Wikipedia’s geographic coordinates for that actor if they fell outside the bounding box.

### Data limitations

Our dataset is limited by several constraints. First, data are self-reported by subnational actors that participate in transnational climate initiatives. While there are most certainly subnational governments outside of these initiatives that are taking climate actions, we are unable to systematically capture and assess them due to the unwieldy nature and infeasibility of collecting every instance outside of common networks and reporting platforms we used in Online-only Table 1^14^. Second, our data collection efforts were primarily limited to English-language sources, although there are certainly non-English language websites and data sources we can research for future iterations of the database. The use of translation websites and application programmer interfaces (APIs) and social media text mining, combined with natural language processing (NLP) techniques, could also aid in further expanding the ClimActor database to include more non-English actors and those not currently reporting to the largest and most common networks available.

## Usage Notes

We developed the ClimActor R package to assist researchers in utilizing our subnational climate action database alongside other datasets. For example, researchers may have data on a subset of subnational actors’ carbon emissions or GDP data that they wish to analyze alongside our dataset. Ensuring actors’ names are consistent (i.e., New York could refer to either New York City or New York state), “clean” (i.e., one dataset could refer to Dallas, TX as simply “Dallas” or “City of Dallas” while referring to the same actor), and disambiguated (i.e., the city of London could refer to London, Ontario in Canada, London, Kentucky in the United States or London, United Kingdom) can often present challenges in analysis. Data from different languages also may include character encodings, accent marks, or various spellings (e.g., Venice and Venezia), which the ClimActor package assists in disambiguating and standardizing. The ClimActor package streamlines the data cleaning process by:

- providing a “key dictionary” of consistent subnational actor names and contextual information (e.g., population, geographical coordinates, etc. whenever these data were available; see above Data Records);
- providing a “country dictionary” of consistent country names (i.e South Korea could also be referred to as “Korea” or “Republic of Korea”; see above Data Records);
- a set of basic data preparation functions to ensure that the dataset in question has the columns and entries needed to work with the subsequent standardization functions;
- a set of data standardization functions to ensure standardized versions of accessory information (entity type, country, ISO code) that allows further functions to standardize actor names;
- a set of string matching functions (using a mixture of exact, phonetic, and fuzzy string matching) to standardize actors’ names based on our collected database of subnational actors;
- a set of post-cleaning functions to update and expand the key dictionary (with novel actors in the user’s database) and to include contextual information (i.e., data records listed in Online-only Table 2) from our database to the user’s dataset.

The string matching functions are based on phonetic matching algorithms and string distance measures compiled in the phonics R package^31^ and stringdist R package^32^ respectively. The process reduces a character string (i.e., a subnational government actor’s name) to a symbolic representation that approximates its English pronunciation before comparing string distance between these measures. This reduced form is then used for matching and avoids complications when there may be common words that increase the length of an actor’s name (i.e Prefeitura de Pedreira and Prefeitura de Pedra Bela) or special characters such as accent marks that may complicate fuzzy string matching^33^. Five different phonetic matching algorithms are used in the package (Metaphone, Nysiis modified, Onca modified refined, Phonex, and Roger Root). These algorithms were selected after evaluating all 16 (nine core and seven modified) that were included in the phonics R package and selecting the five that consistently yielded the most accurate match results in our validation. To validate the phonetic algorithms, the 16 different phonetic representations were first created for all entries in the ClimActor key dictionary. Representations were then visually inspected for duplication, before string similarity measures (based on the average of the Full Damerau-Levenshtein distance, q-gram distance, cosine distance q-gram profiles, Jaccard distance between q-gram profiles, and the Jaro-Winker distance) were used to calculate string similarities between alternative names for entries in the key dictionary (See Table 1 for an example). For detailed descriptions of these algorithms and their respective trade-offs, see Howard (2019)^31^.

**Table 1:** Different Phonetic Representations of alternative names for New York City, NY (“City of New York” and “New York”) and their similarity scores.

Phonetic Algorithm Name	Phonetic Representation for “City of New York”	Phonetic Representation “New York”	Similarity Score
Caverphone	STYFNW	NWYK11	0.38
Caverphone Modified	STFNWK1111	NWK1111111	0.68
Cologne	8236374	6374	0.65
Lein	C142	N350	0
Metaphone	STYFNYRK	NYRK	0.60
New York State Identification and Intelligence System (Nysiis)	CATYAF	NAYARC	0.57
Nysiis Modified	CATAFN	NARC	0.49
Oxford Name Compression Algorithm (Onca)	C315	N620	0
Onca Modified	C315	N620	0
Onca Refined	C306	N809	0.28
Onca Modified Refined	C306	N809	0.28
Phonex	C315	N200	0
Roger Root	182	2470	0.15
Soundex	C315	N620	0
Soundex Refined	C306028093	N8093	0.51
Statistics Canada Name Coding	CTFN	NWRK	0.13

**Fig. 4:**
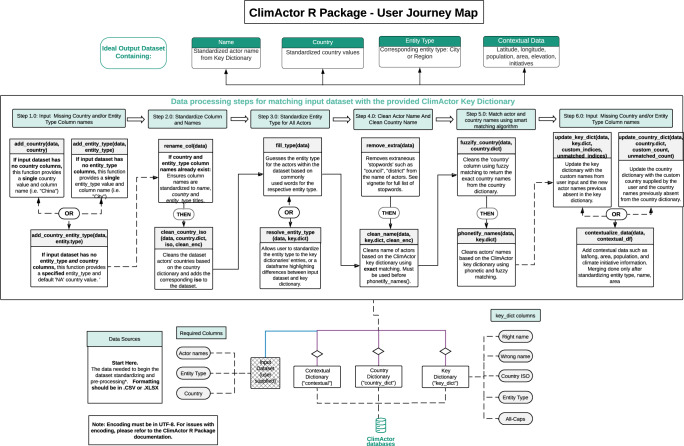
Map of the ClimActor R package that describes the process of matching and combining a user’s dataset with the ClimActor database.

A more detailed user guide outlining the workflow of using the ClimActor package is visible in Fig. 4 and also available at: https://github.com/datadrivenenvirolab/ClimActor. Because the landscape of subnational climate action changes almost daily, the online repository will be updated periodically.

## Data Availability

Data and code for the ClimActor R package functions is available on GitHub: https://github.com/datadrivenenvirolab/ClimActor.

## Online-only Tables

**Online-only Table 1:** Subnational climate action networks and reporting platforms collated for the ClimActor dataset.

Climate Action Platform	Data Source
Alliance of Peaking Pioneer Cities of China	Alliance of Peaking Pioneer Cities of China. (2016). http://www.huanjing100.com/p-1307.html (accessed July 2020).
C40 Cities for Climate Leadership	C40 Cities for Climate Leadership. https://www.c40.org/cities (accessed July 2020).
ICLEI Local Governments for Sustainability carbon*n* Climate Registry	ICLEI Local Governments for Sustainability carbon*n* Climate Registry. www.carbonn.org (accessed July 2020).
CDP Cities	CDP. (2019). 2018–2019 City-wide Emission Reduction Activities. www.data.cdp.net (accessed July 2020).
Compact of States and Regions (currently States and Regions Annual Disclosure)	Compact of States and Regions. (2018). 2017 States and Regions Climate Actions. www.data.cdp.net (accessed July 2020).
EU Covenant of Mayors for Climate and Energy	EU Covenant of Mayors for Climate and Energy. https://www.covenantofmayors.eu/about/covenant-community/signatories (accessed July 2020).
Under2 Coalition	Under2 Coalition (Secretariat The Climate Group). www.under2mou.org (accessed June 2020).
Global Covenant of Mayors for Climate and Energy	Global Covenant of Mayors for Climate and Energy. www.globalcovenantofmayors.org (accessed July 2020).
US Climate Alliance	U.S. Climate Alliance. Inventory of Climate and Clean Energy Policies. Accessed July 2019 from: https://www.usclimatealliance.org/state-climate-energy-policies (accessed July 2020).
Global Climate Action Portal	United Nations Framework Convention on Climate Change. (2020). Global Climate Action Portal (NAZCA). www.climateaction.unfccc.int (accessed July 2020).
US Climate Mayors	US Climate Mayors. www.climatemayors.org (accessed July 2020).
We Are Still In	We Are Still In. (2019). https://www.wearestillin.com/ (accessed July 2020).

**Online-only Table 2:** Data fields and sources included in the ClimActor datasets.

Dataset	Variable name	Description	Source
contextuals	lat, lng	latitude and longitude of the actor’s location, in decimal degrees	Wikipedia
	area	the actor’s land area in sq. km	Wikipedia
	region	one of eight World Bank regions (Asia, Sub-Saharan Africa, Europe, Eastern Europe and Central Asia, Latin America and Caribbean, Middle East and North Africa, North America, East Asia and the Pacific, South Asia)	World Bank. (2018). The world by region. SDG Atlas. Available: http://datatopics.worldbank.org/sdgatlas/the-world-by-region.html.
	state	administrative unit below national government (i.e., if actor is New York City, New York would be listed here)	Wikipedia and Maxmind (2020). Geolite2 Database. Available: https://dev.maxmind.com/geoip/geoip2/geolite2/#License
	admin_1	higher administrative jurisdiction for overlap calculations that is one level above the actor’s level (i.e., if actor is Manhattan, New York City would be listed). Not applicable (NA) for Regions and actors whose higher administrative level would be equivalent to state or country.	Wikipedia
	population	in persons	Self-reported by subnational actors to climate action reporting platforms or Wikipedia
	population_year	year of population data, if reported	Self-reported by subnational actors to climate action reporting platforms or Wikipedia
	initiatives_committed	climate initiatives the actor has committed to	Climate action platforms (see Online-only Table 1)
	num_commit	the number of initiatives an actor participates in	Climate action platforms (see Online-only Table 1)
	initiative columns; APPC, ClimateMayors, ClimateAlliance, NAZCA, etc.	whether a subnational actor participates (1) or does not (0)	Climate action platforms (see Online-only Table 1)
key_dict	right	standardized version of the actor names	Authors
	wrong	unstandardized version of actor names	Various, compiled from various datasets and sources.
	iso	3-letter country code for countries	International Standards Organisation (ISO). (2020). ISO 3166. Available: https://www.iso.org/obp/ui/#search
	entity_type	actors’ entity type (City or Region)	Various, compiled from various datasets and sources and simplified as follows: Regions: states, provinces, prefectures, etc. City: municipality, town, county, comune, district, etc.
country_dict	right	standardized version of country names	Authors
	wrong	unstandardized version of country names	Various, compiled from various datasets and sources.
	iso	3-letter country code	International Standards Organisation (ISO). (2020). ISO 3166. Available: https://www.iso.org/obp/ui/#search
	iso2	2-letter country code	International Standards Organisation (ISO). (2020). ISO 3166. Available: https://www.iso.org/obp/ui/#search
	region	one of eight World Bank regions (Asia, Sub-Saharan Africa, Europe, Eastern Europe and Central Asia, Latin America and Caribbean, Middle East and North Africa, North America, East Asia and the Pacific, South Asia)	World Bank. (2018). The world by region. SDG Atlas. Available: http://datatopics.worldbank.org/sdgatlas/the-world-by-region.html.
	Landarea	country’s land area in sq. km	World Bank. (2018). Land area (sq. km). Available: https://data.worldbank.org/indicator/AG.LND.TOTL.K2.
	Population	in persons	World Bank. (2018). Population. Available: https://data.worldbank.org/indicator/sp.pop.totl.
